# A Theory and Evidence-Informed e-Cycling Intervention for Individuals Diagnosed With Cancer: Development Study

**DOI:** 10.2196/54785

**Published:** 2024-08-16

**Authors:** Jessica E Bourne, Paul Kelly, Miranda E G Armstrong

**Affiliations:** 1 Bristol Medical School Population Health Sciences University of Bristol Bristol United Kingdom; 2 School of Health and Exercise Sciences University of British Columbia Kelowna, BC Canada; 3 Physical Activity for Health Research Centre University of Edinburgh Edinburgh United Kingdom; 4 Centre for Exercise, Nutrition and Health Sciences University of Bristol Bristol United Kingdom

**Keywords:** prostate cancer, breast cancer, electrically assisted cycling, physical activity promotion, behavior change techniques, BCTs, Behaviour Change Wheel, Medical Research Council, Theoretical Domains Framework, TDF, physical activity, e-cycling intervention, e-cycling, cancer, risk of disease, all-cause mortality, behavioral health, instructor, instructors, cancer survivor, patient with cancer, healthy lifestyle, intervention, physical fitness, exercise

## Abstract

**Background:**

Physical activity engagement following a cancer diagnosis is positively associated with survival, reduced risk of disease recurrence, and reduced cancer-specific and all-cause mortality. However, rates of physical activity engagement are low among individuals diagnosed with and being treated for breast cancer or prostate cancer.

**Objective:**

The purpose of this study was to describe the systematic process of developing an e-cycling intervention aimed at increasing physical activity among individuals living with prostate cancer or breast cancer and outline the key components to be implemented.

**Methods:**

The Medical Research Council guidance for developing complex interventions and the Behaviour Change Wheel were used to guide intervention development. Information was gathered from the literature and through discussions with end users to understand factors influencing e-cycling. These factors were mapped onto the Theoretical Domains Framework to identify potential mechanisms of action. Behavior change techniques were selected from theory and evidence to develop intervention content. Interested parties, including cycling instructors, end users, and behavior change experts, reviewed and refined the intervention.

**Results:**

Anticipated barriers and facilitators to e-cycling engagement were mapped onto 11 of the 14 domains of the Theoretical Domains Framework. A total of 23 behavior change techniques were selected to target these domains over 4 one-to-one e-cycling sessions delivered by trained cycling instructors in the community. Cycling instructors were provided a 3-hour classroom training session on delivering the intervention and a 3-hour practical session with feedback. The outcome of this work is a theory and evidence-informed intervention aimed at promoting e-cycling behavior among individuals being treated for breast cancer or prostate cancer, which is currently being implemented and evaluated.

**Conclusions:**

Transparent intervention development and reporting of content is important for comprehensively examining intervention implementation. The implementation of this intervention package is currently being evaluated in a pilot randomized controlled trial. If the intervention is found to be effective and the content and delivery are acceptable, this intervention will form a basis for the development of e-cycling interventions in other survivors of cancer.

**Trial Registration:**

ISRCTN Registry ISRCTN39112034 https://www.isrctn.com/ISRCTN39112034; and IRSCTN Registry ISRCTN42852156; https://www.isrctn.com/ISRCTN42852156

## Introduction

Globally, cancer is one of the leading causes of mortality [1]. Specifically in the United Kingdom, prostate cancer and breast cancer are the most common male and female cancers, respectively [2]. Physical activity engagement following a cancer diagnosis is positively associated with survival, reduced risk of disease recurrence, and reduced cancer-specific and all-cause mortality [3-10]. Furthermore, physical activity engagement during cancer treatment positively impacts quality of life and is associated with reduced fatigue, a common side effect of treatment [11-14]. Despite these positive benefits, rates of physical activity engagement are low among individuals diagnosed and being treated for breast cancer and prostate cancer [15-17], with rates decreasing following diagnosis and during treatment [18,19]. The extent to which individuals diagnosed with cancer are willing to engage in physical activity varies greatly due to differences in the type of treatment, the time scale of treatment, and the number and severity of mental and physical side effects resulting from treatment including fatigue and depression [7,20]. In addition, lack of equipment or facilities as well as lack of time, motivation, and confidence are common barriers to physical activity engagement in this population [21-25]. The lack of clinical guidance on appropriate physical activity to undertake and a limited clinical emphasis on the importance of engaging in physical activity during this time are also barriers to engagement [26].

There is a need for novel interventions to encourage the initiation and maintenance of physical activity in this population. Electrically assisted bicycles (e-bikes; also known as pedelecs) have been highlighted as a potential means through which to increase physical activity, particularly among inactive and older individuals [27,28]. Despite the electrical assistance, e-cycling engagement provides physical activity of at least a moderate intensity [29,30] with the potential to positively impact physical and mental health outcomes [27]. Furthermore, e-cycling has been reported to be an enjoyable activity, an affect response that is considered important for the long-term sustainability of physical activity behavior [31]. To date, the use of e-bikes to increase physical activity in individuals being treated for cancer has yet to be explored.

Developing effective interventions and associated implementation strategies requires an understanding of the target behavior and the factors that influence engagement in that behavior [32]. Specifically, the intervention design and selection of active ingredients with the potential to bring about behavior change should be guided by theory and the context in which the intervention is to be delivered [33,34]. To date, the majority of e-cycling interventions have involved the provision of an e-bike; however, no additional behavior change mechanisms have been reported [35-37]. While the provision of an e-bike provides the opportunity to ride, it may not be sufficient to encourage sustained behavior change [32]. The inclusion of theory-driven behavioral support can help increase the effectiveness of physical activity interventions [38] and engagement with e-cycling in a real-world setting [39]. A recent e-cycling intervention delivered to individuals with type 2 diabetes (T2D; PEDAL2) incorporated behavioral counseling components and demonstrated the potential to improve cardiorespiratory fitness and mental and physical quality of life [40]. The development of the PEDAL2 behavioral counseling was guided by qualitative interview findings in the same population following an e-bike loan [41,42]. While informative, these interviews were designed to assess individuals’ ability to manage their diabetes rather than factors associated with e-cycling engagement [42]. Building on this, qualitative interviews with PEDAL2 participants were conducted after the intervention to ascertain specific barriers and facilitators to e-cycling engagement, and an associated conceptual model was developed [43]. This conceptual model provides a starting point from which to design future e-cycling interventions in other clinical populations. In addition to using this conceptual understanding, the end users of an intervention should be involved in the design of an intervention and implementation strategies to determine factors specific to the population in which the behavior change is targeted [44].

The aim of this study was to develop and refine a theory and evidence-based intervention and associated implementation strategies to promote e-cycling engagement in individuals with prostate cancer or breast cancer (the intervention was named CRANK). The development of the CRANK intervention was guided by formalized intervention development approaches, specifically the Medical Research Council (MRC) guidance for developing and evaluating complex interventions [34] and the Behaviour Change Wheel (BCW) [32], drawing upon the Theoretical Domains Framework (TDF) [45] and stakeholder input [33].

## Methods

### Design

**Figure 1:**
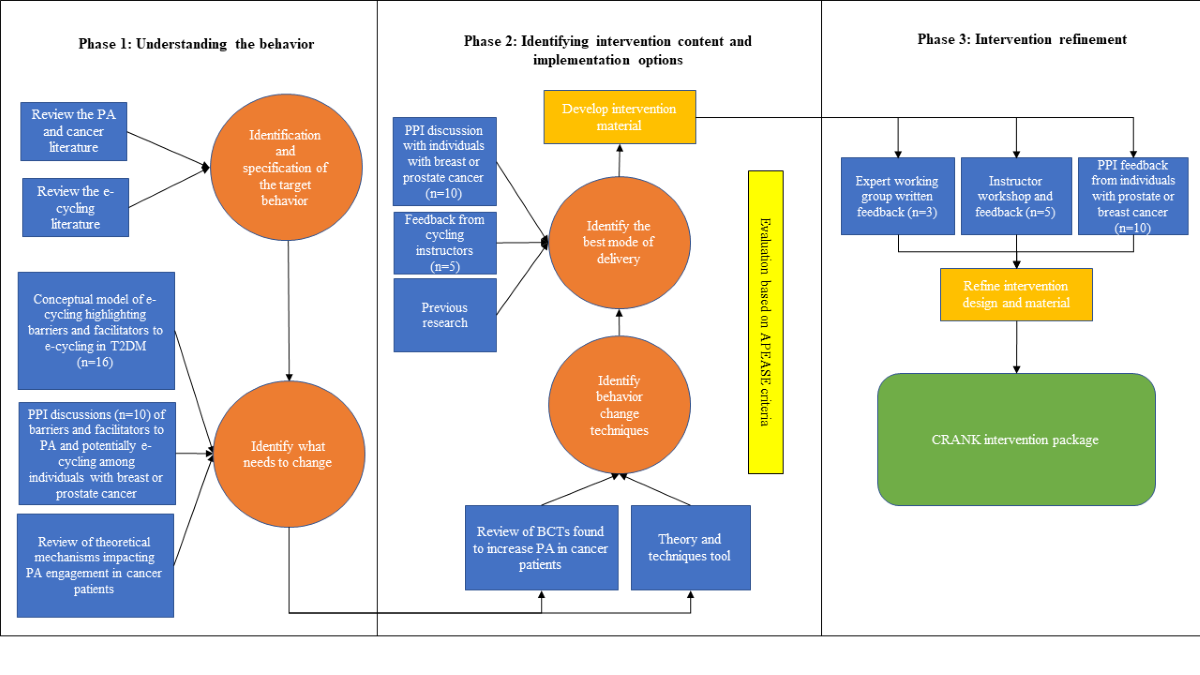
Outline of the CRANK intervention development. APEASE: Acceptability, Practicability, Effectiveness, Affordability, Side-Effects, and Equity; BCT: behavior change technique; PA: physical activity; PPI: patient and public involvement; T2DM: type 2 diabetes mellitus [40,43,50].

The MRC guidance emphasizes the incorporation of both theory and best available evidence to develop complex interventions [34], while the BCW provides a systematic process through which to develop such interventions by completing a series of activities in stages [32]. This research was guided by stage 1 (understand the behavior) and stage 3 (identify content and implementation options) of the BCW. Stage 2 (identify intervention options) was not conducted, as an appropriate behavior change strategy was identified in phase 1 (stage 1 of the BCW guidance), which was directly mapped to behavior change techniques (BCTs) in phase 2 (stage 3 of the BCW guidance). In phase 3, the intervention and implementation strategies were refined through engagement with patient and public involvement (PPI) group members, cycling instructors, and experts in the field of behavior change. Figure 1 [40,43,46] outlines the process of intervention development. PEDAL2 interviews were conducted between August 2019 and November 2020, and the findings from these interviews and the development of the conceptual framework are reported elsewhere [43]. Intervention development, including PPI discussions with individuals living with breast or prostate cancer, expert review and feedback, and instructor workshops and feedback, took place between September 2021 and March 2022. Patient partners with breast cancer (n=4) were recruited through an existing patient and partner group established for a wider research program, while patient partners with prostate cancer (n=6) were recruited through a local prostate cancer charity. Instructors (n=5) were recruited through Life Cycle, the community organization involved in delivering an e-cycling intervention to another clinical population (PEDAL2) [40].

### Phase 1: Understanding the Behavior

#### Define the Problem in Behavioral Terms and Select the Target Behavior

Step 1 focused on the specific problem that the intervention was aiming to address: low levels of physical activity in individuals being treated for breast cancer or prostate cancer. The social and environmental contexts in which the behavior occurs and the individual factors that affect physical activity engagement were considered to identify the major barriers and facilitators to increasing physical activity in this population. Following this, the potential ways in which physical activity could be increased in this population by overcoming some of the identified barriers and facilitators to engagement were considered, and this formed the basis from which this e-cycling intervention was conceived and was based on reviews of the literature and previous work conducted by the authors in a different clinical population.

#### Specify the Target Behavior

Upon selection of the target behavior, we specified who needed to perform the behavior, what needed to be done differently to achieve the change, where and when they needed to do it, and how often and with whom.

#### Identify What Needs to Change

To identify what needs to change in the individual or the environment to bring about the desired change in behavior (ie, engagement in e-cycling), we drew from multiple sources of information, as suggested by the MRC guidance to ensure that theory and research evidence identified are relevant to this context [34]. First, a conceptual model identifying barriers and facilitators to e-cycling, guided by the Capability, Opportunity, Motivation-Behavior (COM-B) model, among individuals with T2D was used to identify the factors that impact e-cycling in clinical populations [43]. Given that e-cycling has yet to be explored among individuals living with cancer, this conceptual model provided a good position from which to begin CRANK intervention development. Second, PPI group discussions took place to gain insight into the factors that may specifically impact e-cycling among individuals living with cancer. In total, 2 web-based discussion groups, 1 each for individuals being treated for prostate and breast cancer, were facilitated and lasted approximately 120 minutes. The groups discussed potential factors that could impact cycling in this population, specifically e-cycling. By the end of the discussion, the group had identified several factors that they felt would impact e-cycling engagement and identified the most important factors based on consensus decisions. Third, the literature was reviewed to identify interventions that were deemed to be successful at increasing physical activity engagement in individuals with cancer (specifically prostate cancer and breast cancer). The theoretical underpinnings of these interventions were identified to advance our understanding of the likely mechanisms of change.

Information obtained from these 3 sources was mapped onto the constructs of the TDF, which is an expansion of the COM-B model, to identify the key constructs that need to change to encourage engagement in e-cycling behavior. The TDF is comprised of 14 theoretical domains that summarize the theoretical constructs from 33 theories of behavior change [45]. In line with the MRC guidance, a program theory was developed to present the hypothesized theoretical underpinning of the intervention.

### Phase 2: Identifying Behavioral Content and Implementation Options

#### Identify Behavior Change Techniques

Having hypothesized the theoretical components required to achieve change in the target behavior, intervention content was developed by the selection of BCTs. The 93-item behavior change technique taxonomy (BCTTv1) [47] was used to provide detailed definitions of BCTs. BCTs were chosen from (1) an examination of systematic reviews that have identified specific BCTs that have been shown to be effective at promoting physical activity behavior in adults living with cancer, with a focus on breast cancer and prostate cancer [48,49] and (2) using the theory and techniques’ web-based tool to identify specific BCTs linking the proposed mechanisms of action identified using the TDF that should be targeted in this intervention [46]. The use of these BCTs in this intervention was considered with regard to acceptability, practicality, effectiveness, cost-effectiveness, affordability, side effects, safety, and equity (APEASE [Acceptability, Practicability, Effectiveness, Affordability, Side-Effects, and Equity] criteria [32]).

#### Identify the Best Mode of Delivery

The mode of delivery including the provider, intensity, and duration was based on previous feasibility findings from participants and instructors of PEDAL2 reported by Bourne et al [40] and from feedback from the CRANK PPI group (n=10) and cycling instructors (n=5). Feedback was elicited through web-based open group discussions. The modes of intervention implementation were considered using the APEASE criteria to assess suitability within the constraints and resources of the trial. Intervention materials were created alongside training manuals for cycle instructors.

### Phase 3: Intervention Feedback and Refinement

The intervention was refined following feedback from CRANK PPI members (n=10), instructors (n=5), and the expert group (n=3). Specifically, the intervention material was sent to PPI members initially for review. After review of these documents, group members met on the web, with 3 group discussions for each of the 3 groups, where feedback on the intervention content and delivery method was provided to the research team. All discussions were recorded in order for the researcher to listen back to the discussion, and notes were taken throughout the discussions. These discussions lasted up to 120 minutes. For instructors, the intervention material was sent for review prior to a 2-hour web-based meeting. At this meeting, the research team presented the intervention material, and instructors were asked to provide feedback on both the instructor training and participant intervention material. Any disagreements within the groups were discussed in the session until a consensus was reached regarding the required intervention changes. The intervention was sent to members of the CRANK trial steering committee with expertise in behavior change. These individuals provided written feedback on the intervention content. The information from CRANK PPI members, instructors, and the trial steering committee was collated and reviewed by 2 researchers, and consensus decisions were made on appropriate changes to be made based on the APEASE criteria.

### Ethical Considerations

The National Health Service Health Research Authority Southwest/Central Bristol Research Ethics Committee provided ethics approval to conduct qualitative interviews among individuals living with T2D as part of PEDAL2 (reference 18/SW/0164). While ethical practice was upheld, formal ethical approval was not obtained for these PPI activities, as advised by the National Institute for Health Research. PPI contributors were involved in the design, implementation, and management of the research process itself. Patient partners were informed of what engagement in the PPI activities would entail prior to agreeing to participate. Ethics approval for the pilot randomized controlled trials to evaluate the intervention (named CRANK) was granted by the National Health Service Health Research Authority Dulwich Research Ethics Committee (REC: 22/LO/0036; CRANK-P) and the Nottingham Research Ethics Committee (REC: 22/EM/0010; CRANK-B), and the protocol for this study is reported by Bourne et al [50].

## Results

### Define the Problem and Select the Target Behavior

Justification for this research is provided in the Introduction section and summarized here for completeness. Individuals living with cancer are less physically active than their healthy counterparts [15,17,51,52]. Specifically, it has been estimated that adherence to the physical activity guidelines among individuals living with cancer ranges from 10% to 47% [15,16,52-56]. Engagement in regular physical activity can help recovery from cancer, reduce the side effects associated with treatment, and reduce the chances of recurrence [3,5,10,11,57]. Several physical activity interventions have been developed for individuals living with and recovering from cancer, with varied success [58-61]. Common barriers to engagement in regular physical activity for individuals with cancer include cancer-related physical symptoms (eg, fatigue), lack of equipment or facilities, lack of knowledge of what to do or support or advice on how to engage in physical activity, lack of motivation and time, and low confidence [21-25].

e-Cycling has been identified as an activity that may overcome some of the identified barriers to engaging in physical activity and promote long-term adherence [28]. The potential of e-cycling to increase physical activity in individuals being treated for or recovering from cancer has not been explored but warrants further investigation. As such, the target behavior of this intervention was to increase physical activity specifically through e-cycling in individuals being treated for breast cancer or prostate cancer.

### Specify the Target Behavior

The aim was to increase individual’s weekly physical activity through engagement in e-cycling. No specific weekly e-cycling targets were imposed by the research team, as we wanted participants to have autonomy over their e-cycling goals. The e-cycling behavior will need to be performed by individuals aged 18 years or older with a diagnosis of prostate cancer or breast cancer on a regular basis, whenever possible with the mantra that every move counts. e-Cycling can be conducted for any purpose (ie, exercise, travel, social, and leisure) in an outdoor setting. e-Cycling can be performed alone or with others.

### Identify What Needs to Change

Drawing on the conceptual model developed from PEDAL2 and incorporating information from PPI discussions with patients being treated for breast cancer or prostate cancer, the intervention team identified key mechanisms of action to target in the intervention to bring about engagement in e-cycling (Tables 1-3).

**Table 1:** Behavioral analysis identifying what needs to change to encourage e-cycling within the capability component of COM-B^a^ model, associated behavior change techniques, and how this will be incorporated into the CRANK intervention.

COM-B component and TDF^b^ domains			What is needed for change?		Behavior change techniques		Description of how this will be incorporated into the intervention-implementation strategy
**Physical capability**							
	Skills	Must feel physically capable to e-cycle, despite potential physical limitations		4.1 Instruction on how to perform the behavior 6.1 Demonstration of the behavior 8.1 Behavioral practice or rehearsal 8.7 Graded tasks		Provide instruction on how to ride the bike and instructor to demonstrate the behavior. Prompt individuals to practice riding during training sessions and at home, starting with riding in quiet locations with minimal surrounding risk and building up to busier locations.	
**Psychological capability**							
	Knowledge	Must have the knowledge of how to perform the activity using the correct and safe technique Knowledge of how to ride safely in traffic or through awareness of cycle paths		4.1 Instruction on how to perform the behavior 2.2 Feedback on behavior		Advise the individual on how to ride correctly and how to ride safely in traffic. Provide information on where to ride (eg, where the nearest cycle paths are located and how to ride a specific journey without traffic). Provide feedback on e-cycling behavior regarding safety and route chosen.	
	Memory, attention, and decision processes	e-Cycling must be perceived as not complicated in order to compete with the car		7.1 Prompts or cues 8.4 Habit reversal 11.3 Conserving mental resources		Individuals encouraged to gather and organize all e-cycling equipment ahead of riding in one obvious location to reduce mental resources and prompt the behavior. Individuals encouraged to e-cycle for a journey that would normally be made by the car.	
	Behavioral regulation	Engaging in physical activity is difficult, setting e-cycling targets and monitoring their behavior helps promote engagement		1.1. Goal setting (behavioral) 1.4 Action planning 2.3 Self-monitoring of behavior		Individuals prompted to set their own goals, which they feel are achievable taking fitness levels, readiness to change, and lifestyle into account (goals will be SMART^c^). Individuals prompted to develop specific planning on how they will achieve each goal set (eg, when and where they will e-bike). Individuals encouraged to monitor their activity using a paper logbook or GPS watch.	

^a^COM-B: Capability, Opportunity, Motivation-Behavior.

^b^TDF: Theoretical Domains Framework.

^c^SMART: Specific, Measurable, Achievable, Relevant, and Time-Bound.

**Table 2:** Behavioral analysis identifying what needs to change to encourage e-cycling within the opportunity component of COM-B^a^ model, associated behavior change techniques, and how this will be incorporated into the CRANK intervention.

COM-B component and TDF^b^ domains		What is needed for change?	Behavior change techniques	Description of how this will be incorporated into the intervention-implementation strategy
**Physical opportunity**				
	Environmental context and resources	Provision of maintenance service will encourage activity engagement Perceived access to safe cycling and parking infrastructure Provision of suitable equipment (e-bike itself and accessories)	3.2 Social support (practical) 1.2 Problem-solving 12.5 Adding objects to the environment	Individuals will be provided with the details of an e-bike maintenance service that can be contacted in case of emergency. Individuals will be encouraged to identify common barriers to e-cycling (eg, weather and access to infrastructure) and plan ways to overcome these problems. Instructor to offer practical solutions based on experience and what other individuals have reported. Individuals to be properly fitted with an e-bike, and adjustments to be made by the instructor to ensure the bicycle is comfortable. Individuals to be provided with basic accessories (bicycle lock, helmet, lights, and pannier). Individuals to be provided with maps of cycle routes to outline safe riding routes.
**Social opportunity**				
	Social support	Support from friends and family regarding e-cycling engagement Watching others engage in the activity and having the opportunity to engage with others while riding and with a similar condition will promote engagement	3.1 Social support (emotional) 3.2 Social support (practical)	Individuals encouraged to seek verbal support from friends and family if they are struggling with the behavior. Individuals will be invited to attend group riding sessions. Individuals encouraged to seek practical support from friends and family if they are struggling to engage in the behavior (eg, going on a bike ride with a friend). Instructor to offer verbal and practical support during loan period with riding catch-ups.

^a^COM-B: Capability, Opportunity, Motivation-Behavior.

^b^TDF: Theoretical Domains Framework.

**Table 3:** Behavioral analysis identifying what needs to change to encourage e-cycling within the motivation component of COM-B^a^ model, associated behavior change techniques, and how this will be incorporated into the CRANK intervention.

COM-B component and TDF^b^ domains		What is needed for change?		Behavior change techniques		Description of how this will be incorporated into the intervention-implementation strategy
**Reflective motivation**						
	Belief about capabilities	Confidence to engage in e-cycling Confidence to e-cycle in traffic and on roads	8.1 Behavioral practice or rehearsal 8.7 Graded tasks 15.1 Verbal persuasion about capability 15.3 Focus on past success		Individuals encouraged to practice riding, particularly in areas where they are comfortable to build confidence. Individuals encouraged to build up to riding in areas in which there is traffic. Instructor to encourage individuals and tell them they are capable of engaging in e-cycling during all sessions. Individuals asked to review their logbooks or e-cycling behavior. Instructor to focus on successful e-cycling experiences to provide motivation and encouragement.	
	Belief about consequences	Hold beliefs that engaging in e-cycling will positively impact various facets of physical and mental health Hold beliefs that e-cycling will enable the individual to ride further, longer, and on hiller terrain due to the assistance	5.1 Information about health consequences 5.3 Information about the social and environmental consequences		The instructors will share information with participants about the importance of engaging in physical activity in general and specifically during cancer recovery and the impact this can have on physical and mental health. Instructors will also share information about how the e-bike can enable individuals to ride further, faster, and on hillier terrain than a conventional bicycle and how the e-bike may open up previously unconsidered journeys.	
	Goals	Setting e-cycling targets will encourage engagement	1.1 Goal setting (behavior) 1.5 Review behavior goal		Individuals will decide (with help from the instructor) upon goals for e-cycling. These goals will be recorded in their intervention booklet. These goals will be SMART^c^ in nature and tailored to the individual’s circumstances. Goals will not be prescribed; individuals will be encouraged to think about them for themselves. At the end of each follow-up session, the instructor and individual will review the goals set at the previous session and together will agree to either keep the goal the same, modify the goal, or create a new goal.	
**Automatic motivation**						
	Reinforcement	Creating established routines and habits for e-cycling	7.1 Prompts or cues 10.9 Self-reward		Individuals are advised to prepare for e-cycling ahead of time and leave equipment together in a visible location to prompt engagement. Individuals are advised to reward themselves, primarily through self-praise, for meeting their weekly e-cycling goals or making progress toward them and record this reward.	
	Emotion	Sense of enjoyment associated with e-cycling Reduced fear of riding on roads, in traffic, or with other road users	5.6 Information about emotional consequences 11.2 Reduce negative emotions		Instructor to provide information on the potential positive emotions that can be gained from e-cycling and to discuss how others have felt from e-cycling. Encourage individuals to record how they feel after e-cycling. Individuals encouraged to try out riding in quiet locations to reduce fear response before building up to busier locations.	

^a^COM-B: Capability, Opportunity, Motivation-Behavior.

^b^TDF: Theoretical Domains Framework.

^c^SMART: Specific, Measurable, Achievable, Relevant, and Time-Bound.

In addition, a review of the literature identified the use of Social Cognitive Theory (SCT) and Transtheoretical Model of Behavior Change as appropriate theoretical models to explain why people adopt physical activity behavior, particularly those with breast cancer and prostate cancer [60-63]. The key constructs of these theoretical models were considered when identifying what needs to change for e-cycling to take place. SCT uses the techniques of mastery, vicarious experiences, and modeling to develop skills and build self-efficacy [64]. SCT also highlights the importance of others when changing behaviors. The Transtheoretical Model is a comprehensive model of behavior change [65]. The 10 processes of change focus on “how” individuals change their behavior. In addition, 2 intervening variables of self-efficacy and decisional balance have been identified as impacting movement between the 6 stages of change.

Overall, 11 of the 14 domains of the TDF were identified as needing to be targeted to encourage engagement in e-cycling. These are shown in Tables 1-3 and summarized below. Specifically, ensuring individuals had the physical skill and knowledge to ride the e-bike and navigate traffic was identified as essential for e-cycling engagement, as was having high confidence to ride the e-bike itself and among traffic (belief about capabilities)*.* To compete with the car as a mode of transport, individuals noted that systems must be in place to ensure e-cycling is perceived as the “easy” option (memory, attention, and decision processes) and that establishing a routine was key (reinforcement)*.* In addition, ensuring individuals have the correct equipment and access to a breakdown service would facilitate e-cycling engagement (environmental context and resources). Setting goals, monitoring the process toward these goals (behavioral regulation), and encouraging individuals to seek out support from family and friends (social support) were seen as important factors that will increase the likelihood of e-cycling behavior. Furthermore, holding positive beliefs about the impact of e-cycling both in regard to physical and mental health and social and environmental outcomes (belief about consequences; emotions) was important to influence behavioral engagement.

### Identify BCTs

A total of 23 BCTs linked to the theoretical domains, as identified through the theory and techniques of web-based tool [46], psychological theories, and literature on BCTs effective at increasing physical activity among individuals being treated for breast cancer or prostate cancer were identified as shown in Tables 1-3. There was significant overlap in the BCTs identified as potentially useful to target the underlying mechanisms of change. The techniques fall across 12 of the 16 BCT categories of goals and planning, feedback and monitoring, social support, shaping knowledge, natural consequences, comparison of behavior, associations, repetition and substitutions, rewards, regulation, antecedents, and self-belief.

### Identify the Mode of Delivery

#### Intervention Provider

Community-based cycling instructors were considered the most suitable individuals to deliver the intervention due to their nationally recognized cycle training certification. However, interviews with instructors who delivered the previous PEDAL2 intervention revealed that instructors desired more specific training on the intervention content prior to delivery [40]. As such, this intervention (known as CRANK) will involve 2 face-to-face intervention training sessions (3 hours each) for instructors incorporating both education and practice, designed to increase confidence in delivering the specific intervention content. Specifically, the training will focus on (1) providing education on the importance of physical activity for individuals with cancer and the general physical and mental health benefits of engaging in physical activity, (2) teaching instructors motivational interviewing techniques that can be used during training sessions to engage with participants, (3) providing information on specific intervention content, and (4) practicing intervention delivery through role play. The training will take place in the community at the cycling organization headquarters, a location familiar to the instructors. The training will be run by 2 researchers. Instructors will also receive a training manual outlining intervention content.

#### Intervention Intensity and Duration

For participants, the intervention will involve 2 face-to-face e-bike skill training sessions and behavioral discussions prior to taking the e-bike home, with no longer than 2 weeks apart between training sessions. The 2 training sessions were found to be appropriate for a clinical population engaging in e-cycling as reported by the cycling instructors and so will be incorporated into this intervention [40]. Training sessions will last approximately 2 hours each. Training session 1 will be mandatory for all participants, while session 2 will be optional and based on the needs and desires as perceived by the participant and the instructor. Participants will then receive a 12-week e-bike loan. The cycling sessions will be delivered at a community center where the cycling organization is based. It is situated in a central urban location easily accessible by multiple forms of transport. This location was deemed desirable to the patient group who had spent a considerable amount of time in clinical settings.

During the e-bike loan period, 2 additional face-to-face sessions will be conducted with the instructor, each for approximately 90 minutes. More face-to-face meetings have been incorporated into CRANK based on feedback from PEDAL2 that interacting with the instructor was motivational and made participants feel supported in their e-cycling journey [43]. These additional sessions will occur at a location of the participant’s choice approximately 4 and 8 weeks into the 12-week e-bike loan. More training and support sessions will be offered if the individual has specific concerns about riding.

Throughout the loan period, the cycling organization will provide a callout e-bike maintenance service. If required, participants will be instructed to call the maintenance number, and a mechanic will come and repair the e-bike.

#### Intervention Fidelity

To ensure the intervention is delivered as intended, a series of fidelity check materials will be incorporated into the intervention as proposed by Lambert et al [66]. Specifically, instructors will be provided intervention content training, and an associated instructor manual will be developed. As part of the training, instructors will engage in a series of role-playing activities, which will be observed by the researchers, and feedback will be provided. The purpose of these role-playing activities is to ensure that instructors understand and are able to deliver the proposed intervention content. In addition, researchers will observe a minimum of 2 training sessions with participants at the start of the intervention. During the observations, the researchers will complete observation checklists and will provide feedback to the instructors. Throughout the intervention, instructors will complete session checklists. These checklists provide detailed information about the specific content that is intended to be covered, including BCTs, during each session including the skill level obtained by the participant and discussions that took place. At the end of the intervention, instructors will be invited to participate in qualitative interviews, in which they can share their experiences of delivering the intervention.

To assess the participant’s engagement with the intervention, a workbook has been created, in which the participant can record their goals, barriers faced, and thoughts on the sessions. Participants will also be provided with a wearable activity tracker to record their activity. The degree to which self-monitoring tools are engaged with will be ascertained. In addition, at the end of the intervention, participants will be invited to participate in qualitative interviews, in which they will be asked about the extent to which they engaged in the intervention activities.

### Intervention Feedback and Refinement

The intervention was refined based on feedback from PPI members, cycling instructors, and experts.

#### Instructor Intervention Training

It was felt, by PPI members and instructors, that instructors delivering the intervention would benefit from meeting with individuals with prostate cancer and breast cancer to discuss their lived experience as part of the training package. This will enable the instructors to understand the potential barriers to physical activity that this clinical population faces and thereby increase empathy. Instructors felt that the adaptability of the intervention needed to be made explicit throughout the training in order to meet the needs of the individual. As such, the training was tweaked to ensure that the ability to adapt the program to individual needs was emphasized. Instructors highlighted the importance of being reimbursed for administration time (eg, contacting participants), which was not part of regular cycling lessons, and that this needed to be made explicit in the training manual. This would encourage instructors to spend more time engaging with the individuals. As such, an agreement with Life Cycle was made to allow instructors to bill for administration hours in addition to instruction hours.

Instructors felt that it was important to provide sufficient training on how to conduct the behavioral counseling component of the intervention in order to increase their confidence and build buy-in from instructors for this component. To ensure sufficient time was given to review and practice these behavioral components, the second training session was extended by 1 hour. In addition, the ability to trial the self-monitoring tools as part of the training was deemed essential prior to instructing participants on how to effectively use these tools. To address this need, all self-monitoring tools were provided to the instructors prior to delivering CRANK for familiarization. The research team answered any questions or concerns about these devices. The instructors also commented that having allocated time to connect with other instructors, also delivering CRANK, was deemed important to share experiences through the incorporation of peer support sessions. As such, bimonthly instructor peer support sessions were specified.

#### Intervention Content and Delivery Mode

Instructors felt that the behavioral counseling should occur at the end of an e-cycling skills training session in a location that was comfortable for both the participant and the instructor (eg, a seated location) rather than trying to incorporate such discussions during skills training. Instructors felt that this would encourage participants to engage more with the behavioral techniques (eg, setting of goals and action planning) and would not become an “inferior add-on” to teaching participants the skills of e-cycling. This was echoed by members of the PPI group who emphasized that these discussions should occur after skill training, enabling participants to think about the information they are receiving and complete the workbook.

The ability to connect with others, with the same diagnosis, was also deemed as highly important to this group. As such, while general group rides were important, having group rides just for individuals with prostate cancer and breast cancer, separately, was seen as potentially more important. These clinical group–specific rides were seen as an opportunity to connect with others in a similar situation, which could help increase motivation and feelings of social support. As such, clinical group–specific rides were incorporated into the intervention. In addition, a WhatsApp group will be formed for the different clinical groups. The ability to connect with others in a similar situation was highlighted as being of great importance, particularly for male participants.

Members of the PPI group felt that support and practice were needed to encourage participants to engage in self-monitoring and that time to practice should be built into training to increase engagement with this technique. In addition, members of the PPI group identified the potential option of using mobile apps to plan routes, in addition to paper maps.

**Figure 2:**
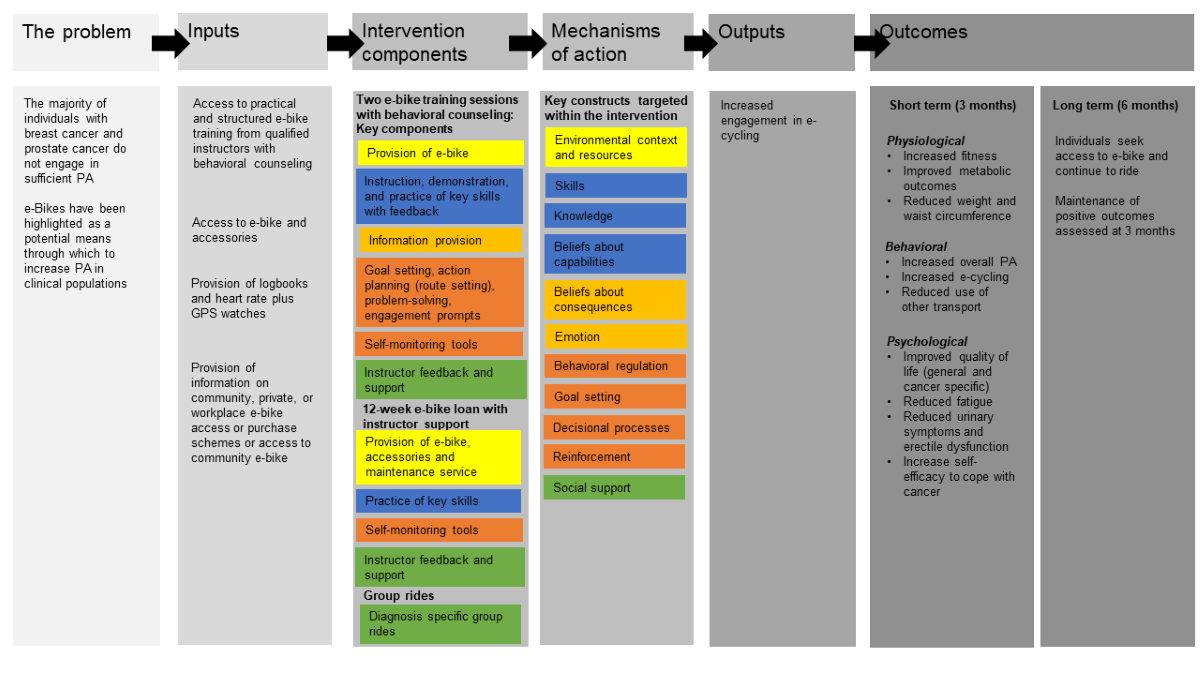
CRANK logical model. The constructs of the TDF targeted in this intervention are color-coded to the intervention components used to target them. PA: physical activity; TDF: Theoretical Domains Framework.

These changes were considered and incorporated into the intervention material. The final program theory, including mechanisms of action and delivery mode, is provided in a logic model in Figure 2.

## Discussion

### Principal Findings

This study reports on the development of a behavioral intervention designed to increase e-cycling behavior in individuals living with prostate cancer or breast cancer. The systematic approach to intervention development involved drawing on theory, evidence, and end-user insights to identify appropriate factors to target in this intervention. It is believed that this process will increase the potential efficacy of the intervention and will allow for an in-depth evaluation of the intervention content to gain an understanding of the intervention effects.

In total, 11 of the 14 domains of the TDF, covering all components of the COM-B model, were found to be relevant to increasing physical activity behavior through e-cycling among individuals living with prostate cancer or breast cancer. Constructs of the TDF not targeted in this intervention were professional or role identity, optimism, and intentions. The 11 TDF constructs identified will be targeted through the use of 23 BCTs from 12 overarching BCT categories [47]. The categories of BCTs selected for use in this intervention are similar to those identified by McVicar et al [67] in their development of an e-cycling intervention for overweight and obese adults using participant workshops. In this intervention, the categories of rewards and regulations were incorporated, which were not part of the intervention developed by McVicar et al [67]. Specifically, in this intervention, individuals were encouraged to reward themselves, primarily through self-praise if they met, or made progress toward, their e-cycling goals. The use of self-rewards has been associated with sustained physical activity behavior at least 6 months after intervention [68]. In addition, participants were encouraged to prepare cycling equipment ahead of time to reduce stress and increase the likelihood of e-cycling over using a motorized vehicle. Ways to assist with behavioral regulation were included in this intervention, as interviews with PEDAL2 participants revealed that trying to remember everything needed for a commute via e-bike was stressful and decreased the likelihood of riding. Overall, McVicar et al [67] identified 16 BCTs for inclusion in their intervention, while the current development process identified 23, of which 12 overlapped. The additional 11 unique BCTs used in this intervention were likely due to the incorporation of sources of information that identified the mechanisms of action that broadly impact PA behavior in the current clinical population as well as those that impact e-cycling specifically. For example, BCT 15.3 focus on past success was incorporated as this technique directly aligns with bolstering self-efficacy, a key component of SCT [64], which has been used in previous physical activity interventions among individuals living with cancer. In addition, BCT 6.5 information about emotional consequences was included due to the conceptual model of e-cycling engagement among individuals with T2D and the finding that individuals are more likely to engage in e-cycling because it is perceived as enjoyable [43]. The BCT 2.3 self-monitoring was found to be an important component to prompt behavior change from a theoretical perspective, from the PEDAL2 conceptual model, and based on discussions with end users and was therefore included in this intervention.

Several of the techniques identified for use in this intervention align with action types identified by Kelly et al [69] in a scoping review of 145 initiatives reporting on intervention content, at the organization and individual levels, aimed at increasing cycling behavior. The review identified commonly used action categories, which will also be used in this intervention. These include knowledge of the benefits of cycling and cycle safety and route planning (ie, education), practical cycling training courses (ie, training), provision of bike accessories and bikes, and access to bike maintenance services (ie, enablement). While the review identified a series of actions associated with restructuring the environment, the majority of these were not suitable for this intervention (eg, provide bike storage facilities and bike wheel channels on stairs or workplace or organizational policies). These components are likely more achievable for organization-based interventions.

### Strengths and Limitations

The behavioral analysis conducted in this study outlines the systematic process used to develop a theoretical understanding of the behavior we are seeking to impact and the mechanisms that may influence this behavior. From here, we were able to identify the theoretical constructs to target and the techniques through which to target these mechanisms in the current population. This transparent method demonstrates the multifactorial nature of this behavior and the complexity of developing a behavioral intervention. However, documenting this process is important, as it allows others to fully understand how the active ingredients of the intervention were selected.

To gain an in-depth understanding of the behavior of e-cycling and the factors that influence engagement, we drew on theory and literature and engaged stakeholders including individuals living with prostate cancer or breast cancer, cycling instructors, and experts in the field of behavior change. It is hoped that gathering information from multiple sources to guide intervention development will increase the chances of developing an intervention that can effectively increase physical activity behavior through e-cycling participation.

The BCTs selected to target each TDF construct were selected using the links proposed by the Theory and Techniques tool, which links BCTs and mechanisms of action based on evidence in the literature [70] and expert consensus [71] and triangulation of these 2 processes [46]. The use of this tool is more appropriate than the use of the BCW guidance, which links BCTs to mechanisms of action based on the “most used” techniques [32].

A potential limitation of this intervention development is that the conceptual model used to guide this intervention was based on findings from 1 city, the same city in which this intervention will be delivered (Bristol, United Kingdom). While appropriate for this intervention, mechanisms of change identified and associated intervention active components may not be applicable to individuals from other cities in the United Kingdom or internationally. A second limitation is that one intervention has been designed for 2 clinical populations, specifically breast cancer and prostate cancer. These cancers generally impact different genders, and there is the potential that these individuals have different barriers and facilitators to e-cycling engagement that may not have been parsed out in this process. However, this work included PPI discussions with both individuals living with breast cancer or prostate cancer, and no outstanding differences were noted between the 2 PPI groups.

### Future Research

This process has led to the development of an intervention with associated participant intervention materials to address some of the barriers identified to e-cycling engagement. In addition, an instructor manual has been created to ensure instructors address these barriers and engage in activities that facilitate e-cycling through training and discussion. The intervention package is currently being tested in a pilot randomized controlled trial [50]. Specifically, the feasibility of delivering this intervention and specific BCTs is being assessed through observations of sessions delivered by instructors with feedback as well as intervention checklists completed during each contact session. The frequency with which each BCT is delivered will be determined and reported. Through workbooks and self-monitoring tools, we will be able to establish the extent to which participants engaged with the BCTs. The impact of omission of BCTs can be compared to effectiveness data and may give insight into the potential efficacy of individual or groups of BCTs selected. In addition, qualitative one-to-one interviews will be conducted with instructors and participants to understand the acceptability of intervention delivery and participation. Testing the delivery of the intervention components is a critical part of intervention development to ensure refinements can be made where required prior to full-scale implementation if suitable. The intervention developed, if appropriate, can be adapted to other groups of individuals being treated for different cancers using the most potent BCTs identified.

### Conclusions

This study presents the process of designing a behavior change intervention targeting physical activity behavior using electrically assisted bicycles for individuals living with breast cancer or prostate cancer. The explicit reporting of the development process and program theory with associated intervention content facilitates the application of in-depth evaluation to determine the efficacy of the BCTs included. This evaluation is currently being conducted and will enable future refinement of the intervention as appropriate.

## Abbreviations

APEASE: Acceptability, Practicability, Effectiveness, Affordability, Side-Effects, and Equity
BCT: behavior change technique
BCW: Behaviour Change Wheel
COM-B: Capability, Opportunity, Motivation-Behavior
MRC: Medical Research Council
PPI: patient and public involvement
REC: Research Ethics Committee
SCT: Social Cognitive Theory
T2D: type 2 diabetes
TDF: Theoretical Domains Framework

## Data Availability

Data sharing is not applicable to this paper as no data sets were generated or analyzed during this study.
